# Integrating a Project-Based Learning Methodology into a Department-Wide Quality Improvement Curriculum: A Nine-Year Experience in Pediatric Subspecialty Training

**DOI:** 10.1177/23821205261428927

**Published:** 2026-02-26

**Authors:** Venessa Lynn Pinto, Danny Castro, Constance M Wiemann, Liza Bonin, Cagri Yildirim-Toruner, Nicholas A. Ettinger, Sharada Hiranya Gowda, Joyee Vachani, Mehmet Fatih Okcu

**Affiliations:** 1 Division of Critical Care, Department of Pediatrics, 506057 Baylor College of Medicine, Texas Children's Hospital, Houston, Texas, USA; 2 Division of Adolescent & Sports Medicine, Department of Pediatrics, 506057 Baylor College of Medicine, Texas Children's Hospital, Houston, Texas, USA; 3 Division of Psychology, Department of Pediatrics, 506057 Baylor College of Medicine, Texas Children's Hospital, Houston, Texas, USA; 4 Division of Rheumatology, Department of Pediatrics, 506057 Baylor College of Medicine, Texas Children's Hospital, Houston, Texas, USA; 5 Division of Neonatology, Department of Pediatrics, 506057 Baylor College of Medicine, Texas Children's Hospital, Houston, Texas, USA; 6 Division of Hospital Medicine, Department of Pediatrics, 506057 Baylor College of Medicine, Texas Children's Hospital, Houston, Texas, USA; 7 Division of Hematology-Oncology, Department of Pediatrics, 506057 Baylor College of Medicine, Texas Children's Hospital, Houston, Texas, USA

**Keywords:** Project-based learning, quality improvement QI, curriculum, graduate medical education, experiential learning, subspecialty fellows

## Abstract

**Background:**

Quality improvement (QI) training of subspecialty fellows is an essential component of graduate medical education, yet often delivered in silos within departments through a variety of instructional methodologies.

**Objective:**

We applied project-based learning (PjBL) as a guiding methodology for a QI curriculum for pediatric subspecialty fellows and describe our curriculum development, instructional design, and its implementation.

**Methods:**

We integrated the Institute for Healthcare Improvement (IHI) Model for Improvement with the 6 key features of PjBL: a driving question, defined learning goals, active participation in educational tasks, collaboration among learners, scaffolding with learning technology, and production of tangible artifacts. The curriculum consisted of workshops followed by application of knowledge to clinical problems through QI projects carried out in their clinical areas. The impact of the curriculum was evaluated by sustainability of fellow-led projects and scholarly output.

**Results:**

From July 2014 through June 2023, 487 pediatric subspecialty fellows participated in 106 QI projects; 80 projects (75%) were sustained for at least 6 months and 65 (61%) were sustained 1 year or longer. Thirty abstracts or posters were presented at regional or national meetings from 21 projects across 11 divisions. Results from six projects were published in peer-reviewed manuscripts.

**Conclusions:**

Our QI curriculum offered fellows practical hands-on learning through sustainable QI projects aligned with their clinical practice, using PjBL as an instructional methodology integrated with the IHI Model for Improvement framework.

## Introduction

The landmark report *To Err is Human: Building a Safer Health System* marked a pivotal shift in healthcare, bringing national attention to the importance of quality improvement and patient safety (QIPS).^1^ In response, healthcare organizations and the academic medicine community recognized the need to train both current and future leaders in these areas.^2,3^ More than a decade ago, the Accreditation Council for Graduate Medical Education (ACGME) mandated QIPS training be included in the common program requirements for training programs.^4^ The adoption of milestone-based assessments established QIPS as a competency for determining trainees’ readiness for independent practice.^5^ It was reinforced by several medical boards, such as the American Board of Internal Medicine, which includes QIPS activities as a requirement for Maintenance of Certification.^6^

Numerous articles describe structured approaches to teaching QIPS, with experiential and project-based learning (PjBL) frequently cited in the medical education literature as effective strategies.^7–14^ However, much of this work has focused on describing the implementation of curriculum and methods to engage trainees in QI projects.^7–11^ Few studies assess educational outcomes, and those that do tend to focus on trainees’ knowledge, attitudes, behaviors, or personal experiences and perceptions.^12–15^ To our knowledge, no other studies have specifically evaluated outcomes of PjBL as a guiding pedagogy.^15^

PjBL is an inquiry-based instructional method grounded in situated learning theory, which posits that the most effective learning occurs in authentic, real-world contexts.^16^ Therefore, PjBL served as an ideal methodology for our curriculum, which aimed to provide pediatric subspecialty fellows with practical, hands-on experience in designing and conducting QI projects relevant to their clinical practice. Recognizing the limited evidence on the impact of PjBL in graduate medical education, our aim was to describe how we applied PjBL as a guiding methodology for curriculum development, instructional design, and implementation, within a department-wide QIPS curriculum at a single academic institution.

## Methods

### Educational Context

Our Department of Pediatrics (DOP) trains approximately 200 fellows annually across 18 ACGME-accredited pediatric fellowship programs and 17 additional fellowship programs approved by the Texas Medical Board or Academic Pediatric Association. The DOP established a centralized educational program for subspecialty trainees, known as Fellows College (FC).^17^ Initially, FC offered limited QIPS education, as each subspecialty training program maintained its own curriculum. This siloed approach required individual programs to secure their own QIPS-trained faculty and resources, which was difficult for smaller programs. To address this challenge, we launched a shared QIPS curriculum through FC.

The nature of this paper is the description of a QI curriculum development and implementation. The goal of this curriculum was to centralize and standardize QIPS education, minimize redundancy, and promote resource sharing. A centralized approach promised to foster a collaborative learning community by enabling fellows to learn alongside peers and faculty from other subspecialties with whom they might not otherwise interact. We utilized The DoCTRINE Guidelines to report our innovative QI curriculum (Supplement 1).^18^ This description of our curriculum development and implementation does not include data from individuals and was deemed non-human subject research.

### Program Development

#### Curricular Content

We used a structured approach to curriculum development.^19^ A needs assessment using the triangulation method helped define the purpose, scope, and content, and it informed learning goals and objectives. Triangulation uses multiple methods to capture a comprehensive understanding of learning needs and to cross-validate information.^20^ We applied it by conducting environmental scans, administering questionnaires to faculty and fellows, and assessing current and anticipated learning needs.^19–22^ Sources for the environmental scan included the expertise of FC leadership, a review of relevant literature, requirements from accrediting and professional organizations, institutional and societal guidelines, and input from a dedicated FC QI Curriculum Committee composed of departmental faculty from various disciplines with expertise in QIPS. This assessment was used to prioritize educational content and define learning objectives for each topic. Key content areas included developing a SMART aim, principles of data analysis, and core quality improvement concepts using the Institute for Healthcare Improvement's (IHI) model for improvement.^23^

#### Instructional Design

**Figure 1.**
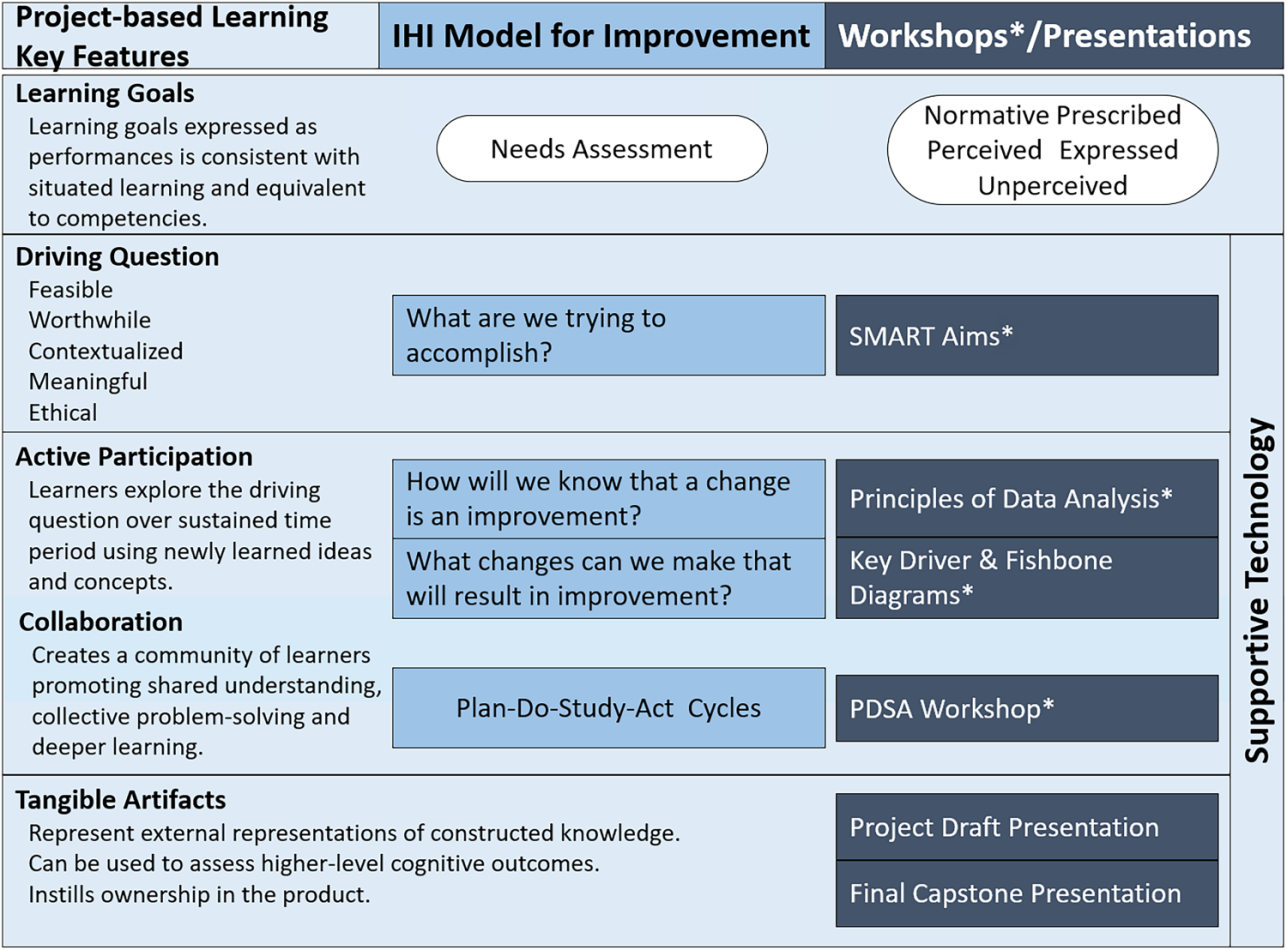
Alignment of key features of project-based learning with the model for improvement framework, along with practical application to QI curriculum sessions. PjBL is an inquiry-based instructional method grounded in situated learning theory that is defined by six key features: a driving question, defined learning goals, active participation in educational tasks, collaboration among learners, scaffolding with learning technology, and the production of tangible artifacts. The IHI model for improvement is a commonly used framework to guide and accelerate improvement work. The model is comprised of three fundamental questions and the Plan-Do-Study-Act (PDSA) cycle. Curriculum sessions comprised of an introductory lecture, interactive workshops and presentation sessions. Workshops included a concise lecture (∼15 min), followed by small-group project work (∼30 min), and concluded with large-group sharing and feedback (∼15 min).

PjBL is rooted in four major theoretical constructs: situated learning, active construction, social interactions, and cognitive tools.^16^ PjBL engages learners in investigating and addressing authentic, real-world problems, thereby cultivating the development of deep, transferable knowledge. It also fosters rich social interactions that build a community of learners who collectively explain, share, and debate ideas as they tackle complex problems. The IHI model for improvement and PjBL features were intentionally aligned across the curriculum, with workshops sequenced to correspond to the three fundamental improvement questions and culminating in tangible project outputs.^23^ This section illustrates how we operationalized this integration, especially when designing and sequencing interactive workshops and learning activities. (Figure 1)

The IHI model for improvement is a commonly used framework to guide and accelerate improvement work. The model is comprised of three fundamental questions that drive improvement and the Plan-Do-Study-Act (PDSA) cycle to test and adapt changes to ensure they result in the desired improvement.^24^ The PjBL pedagogy aligns with recommendations to embed QIPS concepts and competencies into meaningful educational experiences through improvement projects that enhance outcomes for learners, organizations, and patients.^3,25,26^ It is anchored by six key features: a driving question, defined learning goals, active participation in educational tasks, collaboration among learners, scaffolding with learning technology, and production of tangible artifacts.^16^

Establishing explicit learning goals that align with national and accrediting standards is critical for effective implementation of PjBL and as a guard against straying from the required educational outcomes.^16^ The ACGME's standards for QIPS training in graduate medical education and the model for improvement formed our initial learning goals. Our needs assessment further informed our learning goals, as the initial requirements simply stated that trainees must participate in QI activities. Evolving ACGME requirements, the introduction of milestone-based assessments, and increased QIPS exposure during medical school and residency training helped to further refine learning goals and ensure their alignment and relevance.^3,5,13^

The driving question, a central concept, guides instruction and immerses learners in authentic, real-world problems. It aligns with the initial fundamental question posed in the model for improvement: “*What are we trying to accomplish?*”.^24^ Well-crafted driving questions share characteristics with aim statements, which answer this first fundamental question.^16^ Consequently, an initial workshop focused on SMART aim development provided fellows with the opportunity to formulate their own driving questions based on clinically relevant challenges. These questions subsequently guided them in constructing their aim statements.

Workshops balanced hands-on project work with lecture-based instruction to promote application of new knowledge and learners’ engagement, incorporating the PjBL feature of active participation.^16^ The project work allowed teams to apply newly acquired concepts directly to their projects, while large group discussions at the end of workshops integrated collaboration, which aimed to build shared understanding and foster deeper learning. Didactics, which were intentionally sequenced to align with the IHI model for improvement, began with the introduction of basic QIPS concepts and the IHI model for improvement.

The SMART aims workshop, which aligned with the first fundamental question, followed this session. The Principles of Data Analysis workshop aimed to teach learners how to identify ideas for change and select appropriate measures—quantitative and qualitative—to evaluate whether those changes led to improvement. It directly addressed the remaining two questions of the model for improvement: “*How will we know that a change is an improvement?*” and “*What changes can we make that will result in improvement?*”.^23^ The PDSA workshop concluded the didactic series as learners began planning how to implement their testable ideas for change.^24^ (Figure 1)

The creation of tangible artifacts addresses the driving question and is a feature of PjBL that was integrated into our curriculum through two sessions: teams presented draft projects for critique and then shared results after implementation. The former provided a platform for valuable formative feedback from peers, coaches, and faculty, enabling teams to refine their projects before implementation. The latter showcased each team's exploration of their driving question, their mastery of curriculum learning goals, and the production of assessable work products. In both sessions, teams presented and commented on each other's work, fostering a collaborative learning community that shared understanding and collective problem-solving. It further reinforced active participation and collaboration, which are hallmarks of PjBL.

Technological support of learning is a key aspect of PjBL, as it empowers learners to access, gather, analyze, and share data. Moreover, it fosters connections and collaborations within and beyond educational settings and enables learners to create artifacts.^16^ The introduction of available technological tools and resources to assist with specific tasks was incorporated into the relevant workshops. Technology provided flexibility during and immediately after the COVID-19 pandemic as we incorporated virtual workshops with breakout rooms followed by a hybrid format. A library of recorded sessions was also made available for asynchronous learning. This approach demonstrated the use of technology to sustain collaboration and connection.

### Program Evaluation and Evolution

We evaluated the curriculum through three key metrics: the number of projects initiated within the clinical learning environment, the sustainability of these projects at 6- and 12-month post-completion, and scholarly output resulting from this work. These measures would align with Level 4 of the Kirkpatrick Model (Results) as system-level outcomes.^27^ Sustainability of projects was self-reported through query of division QI coaches and program directors as to whether or not QI projects were sustained at 6 and 12 months after project implementation. To encourage fellows to think of how they will ensure their project sustains after they have completed their curriculum timeline, project presentation templates were modified to include a slide on sustainability plans. Our academic year (AY) runs from July to June. The last year included, AY 22, was from July 2022 to June 2023. Twelve month sustainability of projects was tracked till June 2024. Divisions were queried and results analyzed late 2024. Scholarly output was included if abstracts were accepted for presentation or manuscripts were published by June 2024.

Aligning learning goals with educational requirements is crucial, but requirements alone may not inspire curiosity and engagement—central tenets of PjBL. Hence, we continually adapted the curriculum to address learning needs identified through changes to requirements, learners’ feedback, curriculum evaluations, and assessments of presentations. In the early years, individual workshops and presentations were surveyed, however in later years, surveys were only sent out to assess the overall curriculum at the end of the academic year. Normative needs, including new milestone requirements and updated common program requirements, and expressed needs, such as learner feedback, indicated a requirement for more breadth and depth in data analysis principles.^4,5,20^ Hence, the content of this workshop was expanded to explain types of measures, selection criteria, and effective data visualization. Additionally, the assessment of artifacts during the presentation sessions enabled us to gauge learners’ comprehension of the content and their evolving understanding over time. This assessment of their unperceived needs guided our decision to include workshops on constructing key driver and fishbone diagrams and executing PDSA cycles.^16,20^

The curriculum content and timeline for academic year 2023–2024 are summarized in Table 1. There was an introductory didactic lecture on QI fundamentals that also addressed how to pick a project. This was followed by monthly workshops on crafting a “SMART aim”, problem analysis with key driver and fishbone diagram, selecting appropriate measures, analyzing and displaying data, and developing PDSA cycles. Workshops included 15 min for didactic teaching, 30 min for small group activities to apply the concept to their individual projects, and 15 min for groups to receive feedback. Fellows then showcased draft project presentations for critical feedback before implementation. After 4 months, they present their final project presentations with results. Participating trainees and coaches provided feedback to groups at each workshop and after presentations. All QI coaches who volunteered for the various groups of pediatric subspecialty fellows were identified by the core curriculum faculty as departmental faculty with QI training and experience. They were expected to regularly meet with their team, guide project development, attend Fellows College sessions, and provide feedback.

**Table 1.** Curriculum Content and Timeline—Academic Year 2023–2024.

2023-2024 Sessions *All Sessions Take Place from Noon- 1pm*		Date
Session 1	Intro to QI/ Description of the Curriculum didactic 45-min session	August 23, 2023
Session 2	SMART AIM Workshop*	September 27, 2023
Session 3	Key Driver and Fishbone Diagram Workshop*	October 17, 2023
Session 4	Measures/Data Display Workshop*	November 30, 2023
Session 5	PDSA Workshop*	December 20, 2023
Session 6	Draft Project Presentations: 3-4 teams per session	Multiple Dates Jan 2024
Session 7	Final Project Presentations with Results: 3-4 teams per session	Multiple Dates May 2024

* Workshops included a concise lecture (∼15 min), followed by small-group project work (∼30 min), and concluded with large-group sharing and feedback (∼15 min).

## Results

**Figure 2.**
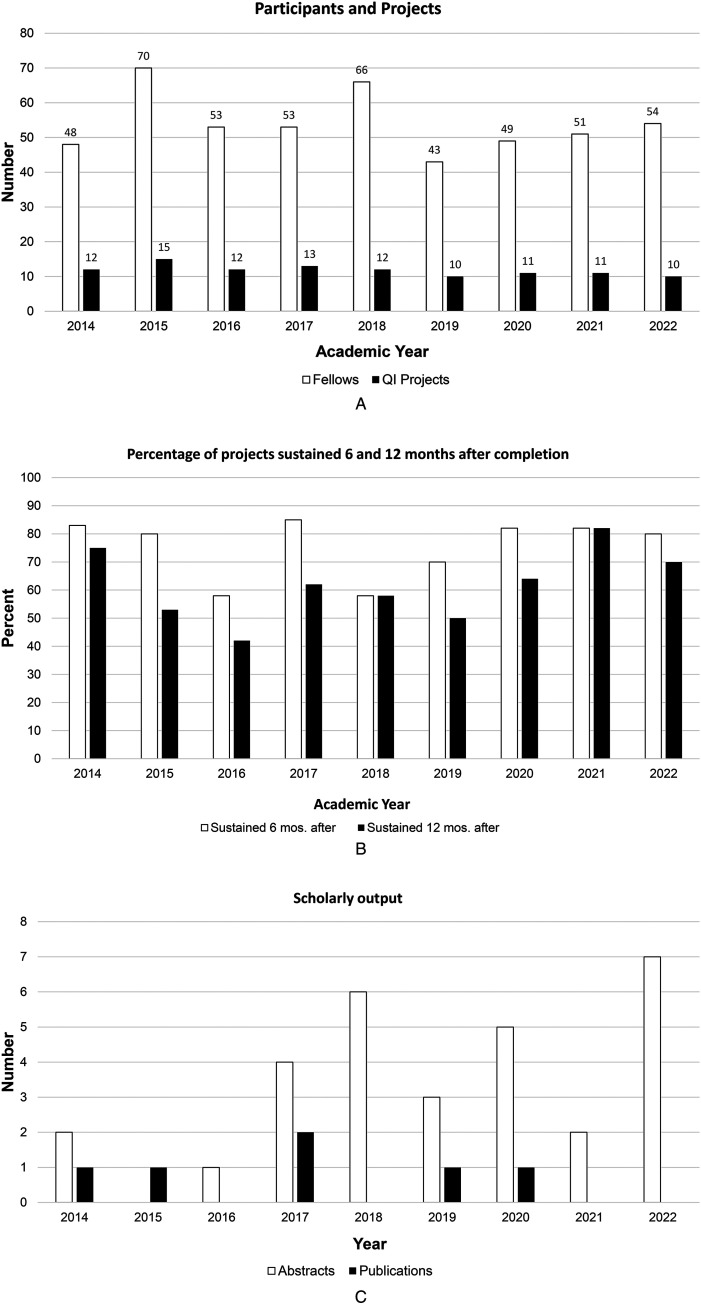
(A) Number of pediatric subspecialty fellows participating in the QI curriculum (white columns) and number of QI projects carried out each year (black column). (B) Percentage of QI projects sustained for at least 6 months (white columns) and 12 months after project implementation (black columns). Last academic year included was from July 2022 to June 2023 with 1 year follow up for sustainability till June 2024. (C) Number of abstracts presented (white columns) and peer-reviewed manuscripts published (black columns) from QI projects within our curriculum by year.

During a 9-year period starting July 2014, 487 pediatric subspecialty fellows participated in 106 QI projects that were implemented in both inpatient and outpatient settings (Figure 2A). On average 54 fellows participated each year, contributing to an average of 12 QI projects a year. Sixteen departmental divisions consistently engaged with the curriculum during this timeframe, with an average of 10 divisions participating per year and 23 faculty coaches supporting the teams annually. Eighty projects (75.5%) were sustained for 6 months and 65 (61.3%) were sustained at least 1 year after curriculum implementation (Figure 2B). Feedback gathered from participants and coaches suggested that changes in clinical practice such as new guidelines or change in management, overlap with ongoing divisional QI and process improvement work, change in institutional priorities, and reliance on manual work that was not automated were various reasons for projects that were not sustained. Scholarly dissemination included 30 abstracts presented at local and national meetings, representing 21 projects from fellows in 11 departmental divisions. Additionally, six projects from four divisions resulted in peer-reviewed publications (Figure 2C).

Overall ratings from surveys on individual sessions in the early years ranged from 4.40/5 in 2015 to 4.95/5 in 2018; survey response rates for sessions ranged from 15% to 65%. Data from 2016 was missing, and 2021 and 2022 survey had free text questions rather than numeric scores. Thematic analysis of comments provided showed that the curriculum's strength lay in team-based project development and real-world implementation. Fellows also appreciated the constructive peer critique received during the presentation of their projects. Common challenges included the limited availability and engagement of some coaches throughout the project's lifecycle and workshop sessions. Time constraints—stemming from condensed training periods and competing clinical responsibilities—also were reported as barriers by some participants. Finally, a minority of fellows considered certain educational content as too elementary for their levels of training.

## Discussion

We describe a structured approach to curriculum design, implementation, and evaluation of a department-wide QIPS curriculum for pediatric subspecialty fellows at a single academic institution. This curriculum offered fellows practical experiences in creating and conducting QI projects aligned with their clinical practice, using PjBL as an instructional methodology integrated with the IHI model for improvement framework. The initiation of at least 10 projects annually, with a majority of them sustained up to 1 year after completion, attests to the success of our curriculum. Dissemination of scholarly products was rare initially but increased over time, specifically in the form of abstracts.

To our knowledge, no published studies have explicitly used PjBL as the central pedagogical method for QIPS education. In fact, several systematic reviews assessing QIPS curricula highlight the substantial heterogeneity in educational content, teaching methods, target learners, and reported outcomes.^28–30^ Although many articles in the QIPS literature describe the integration of adult learning principles such as experiential, team-based, or project-based approaches, they often remain descriptions of structured curriculum design without the use of the six key features of PjBL as a scaffold. Most of the existing research on PjBL originates from K–12 education.^15^ Few studies examine its potential in health professions education, and those that do focus primarily on implementation strategies rather than its effect on educational outcomes.^15^ Systematic reviews indicate that most QI curricula are well-received and enhance learners’ knowledge or confidence in conducting QI work. However, the variability in content and methodology has hindered analysis of the relationship between instructional methodologies and curriculum effectiveness, as well as the synthesis of results.^28,30^ Hence, evidence for more impactful outcomes, such as behavioral change, organizational impact, and patient outcomes, is limited or inconsistent.^28–31^

Our curriculum represents an effort towards the call for curricula that promote and assess frameworks and models integrating QIPS education with clinical care, while evaluating their impact on educational and patient outcomes.^25,30^ Limitations were that our educational outcomes were limited to group assessments and did not include individual assessments of learning. While assessing individual participants was not our primary objective given the team-based nature of the curriculum, it would have been challenging given variable participation by individuals in project work and varying contributions to scholarly output, which could also fluctuate over the curriculum timeline. We also did not collect data related to achievement of project aims, which could have served as surrogates for patient outcomes, or on factors that contributed to individual project success or failure. Another limitation is that we did not directly assess Kirkpatrick Level 3 (behavioral change), and survey data (Level 1-2) were not designed for rigorous outcome evaluation. This limits causal inference regarding the effect of PjBL on individual practice change. Despite these limitations, the growing emphasis on outcomes-based medical education, particularly in QIPS, renders these results valuable in understanding why our curriculum did or did not achieve its intended outcomes.^25,30,32,33^

Measurement of curricular outcomes seeks to answer, “Did it work?”. Yet, it is equally important to understand, “How did it work?”—which requires examining the processes underlying an intervention's effectiveness.^32^ Fellows highly valued the opportunity to apply QIPS principles through team-based project development and real-world implementation, suggesting effective integration of the IHI model for improvement into the PjBL methodology. Participation in the workshop series and project-presentation sessions allowed learners to revisit their driving questions, refine solutions iteratively, apply new concepts cumulatively, and engage in ongoing discussions with stakeholders. This engagement in cognitive processes, such as application, judgment, evaluations, and critical thinking, over time is central to the inquiry-based methodology of PjBL.^15^ Moreover, the model for improvement scaffolds these activities by emphasizing that answering its three questions is iterative, as insights gained from one question or PDSA cycle often reshape thinking in another. Fellows also appreciated the constructive peer critique they received during project presentations. Anecdotal observation suggests that as teams presented and commented on each other's work in both activities, a collaborative learning community emerged that fostered shared understanding and collective problem-solving, reinforcing active participation and collaboration, which are hallmarks of PjBL. However, the low response rates to the surveys sent to fellows and coaches, coupled with the absence of formal analysis of the processes contributing to curricular effectiveness, limit this interpretation to inferred assumptions. Causal attribution of improvement cannot be established in the absence of a comparator curriculum.

This limitation highlights the essential role of conceptual frameworks in medical education.^32^ Conceptual frameworks, derived from educational theories, models, or best practices, provide insights into the success or failure of interventions.^32^ They also offer structured approaches to conceptualize and analyze complex processes such as behavioral change and system-level impact.^32^ Moreover, multiple frameworks may be suitable for a specific situation, each illuminating different aspects of a problem or research question. Consequently, future studies in QIPS education should incorporate conceptual frameworks and explicitly articulate the assumptions and principles underlying those frameworks, which will enable educators to build upon one another's work, advancing the field's collective understanding towards integrating QIPS education with clinical care.^25,32^ Future iterations of this curriculum could further integrate competency-based assessment frameworks, particularly ACGME milestones and entrustable professional activities (EPAs). Although the curriculum was conceptually aligned with QIPS competencies, formal mapping of learner activities and project outcomes to milestone-based assessments was not performed. Incorporating milestone- or EPA-linked assessments could enable more systematic evaluation of learner progression in areas such as systems-based practice and practice-based learning. This approach may also support longitudinal tracking of competence and strengthen alignment with competency-based medical education, while providing clearer evidence of how QIPS curricula contribute to learner development and system-level impact.

## Conclusion

We presented a structured, department-wide QIPS curriculum for pediatric subspecialty fellows, integrating the instructional methodology of project-based learning into the IHI model for improvement framework. For more than 9 years, the curriculum demonstrated sustained implementation of projects and increased scholarly dissemination. Unlike prior QIPS curricula, this program explicitly applied the six core features of PjBL, fostering active, collaborative, and iterative learning. Although limitations included the absence of individual assessments and project outcome data, the curriculum enabled meaningful engagement and real-world application. The authors advocate for future QIPS curricula to incorporate and articulate conceptual frameworks to advance the field. We emphasize the importance of understanding not only whether a curriculum works, but also how and why it works.

## Supplemental Material

sj-docx-1-mde-10.1177_23821205261428927 - Supplemental material for Integrating a Project-Based Learning Methodology into a Department-Wide Quality Improvement Curriculum: A Nine-Year Experience in Pediatric Subspecialty Training Supplemental material, sj-docx-1-mde-10.1177_23821205261428927 for Integrating a Project-Based Learning Methodology into a Department-Wide Quality Improvement Curriculum: A Nine-Year Experience in Pediatric Subspecialty Training by Venessa Lynn Pinto, Danny Castro, Constance M Wiemann, Liza Bonin, Cagri Yildirim-Toruner, Nicholas A. Ettinger, Sharada Hiranya Gowda, Joyee Vachani and Mehmet Fatih Okcu in Journal of Medical Education and Curricular Development
